# Rich Efficient Short-Chain Phthalate Esters-Degrading Bacteria and Their Degradation Mechanisms in Recycled Plastic Wastewater Treatment Plant

**DOI:** 10.3390/microorganisms14091896

**Published:** 2026-08-26

**Authors:** Zhilian Gong, Lingshan Li, Yajie Li, Xiao Zhang, Sidan Gong, Yong Li

**Affiliations:** 1 College of Food and Biological Engineering, Xihua University, Chengdu 610039, China; 2 Faculty of Environmental Engineering, Southwest Jiaotong University, Chengdu 610059, China

**Keywords:** DMP/DBP-degrading bacteria, high-throughput sequencing, substrate profile, whole-genome sequencing, functional gene

## Abstract

Microbial degradation of phthalate esters (PAEs) is regarded as a highly promising green remediation strategy for PAE-contamination, but it still remains constrained by the scarcity of efficient microbial resources capable of degrading PAEs completely under complex conditions. This study systematically explored microbial resources resistant to dimethyl phthalate (DMP)/dibutyl phthalate (DBP) stress along with the putative degradation mechanisms in recycled plastic sewage treatment plants through high-throughput sequencing, DMP/DBP-degrading bacteria screening, and whole-genome sequencing of representative efficient DBP-degrading strains. High-throughput sequencing result revealed substantial enrichment of PAEs-degrading bacteria in activated sludge exposed to 500 mg L^−1^ DMP/DBP. Further analysis demonstrated that the recycled plastic sewage treatment plant harbored rich culturable DMP/DBP-degrading bacteria, comprising 19 genera and 27 species of DMP-degrading bacteria, as well as 11 genera and 20 species of DBP-degrading bacteria. Notably, strain SWLYDMP 33, a potential novel species, along with 11 genera such as *Paenirhodobacter*, *Ciceribacter*, and *Neorhizobium* has been scarcely reported in association with PAEs degradation. Given its affiliation with a dominant genus and its superior broad-spectrum degradation performance, the representative efficient DBP-degrading strain SWLYDBP 51 was selected for whole-genome sequencing. Genome annotation of strain SWLYDBP 51 uncovered a wealth of functional genes implicated in DBP metabolism, and the putative complicated DBP degradation pathways were reconstructed, suggesting that SWLYDBP 51 might possess the genetic capacity for effectively degrading DBP under diverse environmental conditions. Overall, this study provided abundant efficient microbial resources and a theoretical reference for the bioremediation of PAEs contamination.

## 1. Introduction

Phthalate esters (PAEs) are widely used as plasticizers globally, with characteristics such as high lipophilicity and chemical stability. Their water solubility of PAEs varies with the alkyl chain length and typically, short-chain PAEs such as dimethyl phthalate (DMP) exhibit higher water solubility than long-chain ones. PAEs are easily released into the environment and tend to persist due to their affinity to plastics via hydrogen bonds [1,2], which poses significant threats to the ecosystem and human health [3,4]. Consequently, it is urgent to effectively control PAEs contamination.

Microbial degradation of PAEs is widely considered for advantages such as low cost, environmental friendliness, and remarkable sustainability. It involves both the hydrolysis of alkyl ester bonds and the ring-opening cleavage of phthalic acid (PA), and ultimately PAEs are converted into small molecules [5]. Most PAEs-degrading bacteria hydrolyze ester bonds via de-esterification to convert PAEs into PA [6,7,8]. Some strains degrade long-chain PAEs into PA through β-oxidation and transesterification [9,10,11]. PA is then converted to protocatechuic acid (PCA) through a series of reactions including hydroxylation, dehydrogenation, and decarboxylation. Subsequently, PCA is degraded into small-molecule metabolites through aromatic ring cleavage, after which it enters the tricarboxylic acid (TCA) cycle and is completely mineralized [12].

The exploration and application of highly efficient PAEs-degrading bacterial resources are pivotal to the development of microbial-degrading PAEs technology. Various efficient PAEs-degrading strains have been isolated including *Pseudomonas*, *Comamonas*, *Sphingomonas*, *Gordonia*, *Rhodococcus*, and *Bacillus* [13,14,15]. Owing to the variation in environmental conditions and strain characteristics, PAEs-degrading strains had different substrate profiles, degradation capacities, and degradation mechanisms [15]. Some strains only partially degraded PAEs and could not mineralize intermediate products [16,17], whereas others could degrade PAEs completely [12]. Li et al. (2023) found that *Acinetobacter baumannii* DP-2 could mineralize 15 types of PAEs and employed a comprehensive metabolic pathway to convert dibutyl phthalate (DBP) into PA via hydrolysis, β-oxidation, and decarboxylation [18]. Whole-genome analysis of *Priestia megaterium* P-7 isolated from cotton fields revealed that it harbored key degradation genes encoding esterase, decarboxylase, and dioxygenase [19]. Genomic sequencing of *Gordonia* sp. LUNF6 isolated from wastewater demonstrated that it metabolized PAEs via genes such as hydrolases genes, a *pht* cluster and a *pca* cluster [20]. However, current microbial degradation technologies for PAEs still faced such challenges as the shortage of efficient PAEs-degrading microorganisms with broad substrate profiles and the capability of mineralizing PAEs completely under complex environmental conditions. The mechanisms of microbial-degrading PAEs required further study.

Wastewater treatment plants, especially industrial wastewater treatment plants serve as critical points connecting pollution sources with the natural environment and their degradation capacity is vital to regional environmental safety [21]. Microbial communities play a key role in PAEs degradation within wastewater treatment plants [22]. Most previous studies focused on bacterial degradation of PAEs in municipal domestic sewage treatment plants and limited studies were in industrial sewage treatment plants with high concentration of PAEs [15]. High-concentration PAEs created a strong selective pressure which promoted the evolution and enrichment of PAEs-degrading bacteria [15,23]. Recycled plastic wastewater treatment plants, with high concentrations of PAEs, might harbor abundant efficient PAEs-degrading bacteria.

Considering that short-chain PAEs constituted a significant proportion in wastewater treatment plants [24], this study systematically explored microbial resources resistant to high concentrations of DMP/DBP and the degradation mechanisms in a recycled plastic sewage treatment plant by high-throughput sequencing, DMP/DBP-degrading bacteria screening, and whole-genome sequencing of a representative efficient DBP-degrading strain. These findings might provide valuable microbial resources and theoretical reference for microbial-degrading PAEs technology.

## 2. Materials and Methods

### 2.1. Sample Collection

The activated sludge samples used for microbial community analysis and screening of DMP/DBP-degrading bacteria were collected from a recycled plastic wastewater treatment plant in the Southwest Renewable Resources Industrial Park, Neijiang City, Sichuan Province. The DMP/DBP concentrations in the inlet, the outflow and the activated sludge are listed in Table S1. The activated sludge samples were transported back to the laboratory with ice packs.

### 2.2. Microbial Community Under the Stress of High DMP/DBP Concentration and Their Capacity of Degrading DMP/DBP

Twenty-five grams of activated sludge was added to 100 mL of mineral salt medium (MSM) containing 500 P and 500 mg L^−1^ DBP, and then were incubated for 7 days at 28 °C and 200 rpm. The residual concentrations of DMP and DBP in the liquid medium and microbial biomass (OD_600_) were measured every 24 h. The concentrations of DMP and DBP were determined by high-performance liquid chromatography (HPLC) (Waters, Milford, MA, USA), equipped with a C18 reverse-phase column (250 mm × 4.6 mm, 5 μm), as follows: An aliquot of 10 mL of the culture medium was transferred into a separatory funnel, to which 20 mL of ethyl acetate was added. The mixture was shaken vigorously and extracted for 30 min. The organic phase was collected, and the aqueous phase was extracted twice more with fresh portions of ethyl acetate. All organic fractions were combined and evaporated to dryness using a rotary evaporator, and the residue was redissolved and made up to 10 mL with methanol. Subsequently, 1 mL of the resulting solution was filtered through a 0.22 µm nylon membrane filter, and the filtrate was subjected to ultrasonication for 30 min before HPLC analysis [25]. The mobile phase consisted of methanol and water in a ratio of 80:20 (*v*/*v*) with a flow rate of 1.0 mL min^−1^. The detection wavelength, injection volume, and column temperature were set at 228 nm, 20 μL, and 35 °C, respectively. For quantification, an external standard method was employed, which yielded excellent linearity (*R*^2^ > 0.999). To ensure analytical quality, procedural blanks were analyzed to correct background contamination, and spiked recovery experiments were conducted on real samples. The mean recoveries were within 95–105%. The limits of detection and quantification (LOD/LOQ) were 0.05 and 0.15 mg L^−1^, respectively, based on a signal-to-noise ratio of 3:1 and 10:1. After 7 days of incubation, microbial community analysis was performed by high-throughput sequencing of the amplified V3–V4 region of the 16S rDNA using primers 338F and 806R.

### 2.3. Screening and Identifying DMP/DBP-Degrading Bacteria and Their Capacity of Degrading DMP/DBP

To isolate DMP/DBP-degrading bacteria, the culture media above were subjected to the spread plate method on solid MSM containing 500 mg L^−1^ DMP/DBP. To identify the isolated strains, the 16S rRNA gene was amplified by PCR with universal primers 27F and 1492R [26], and sequenced. The resulting gene sequences were then compared against the GenBank database using BLAST v2.13.0.

To determine the DMP/DBP-degrading capacity, the bacterial suspension (OD_600_ = 1) was inoculated at a ratio of 2% into MSM containing 500 mg L^−1^ DMP/DBP, and incubated for 7 days at 28 °C and 200 rpm. The residual DMP/DBP content in the medium was determined by HPLC. An uninoculated liquid MSM containing 500 mg L^−1^ DMP/DBP was used as a blank control.

### 2.4. Determination of the Substrate Profile of Representative Efficient DBP-Degrading Bacteria

To determine the substrate profile, the representative efficient DBP-degrading bacterial suspension (OD_600_ = 1) was inoculated at a 2% ratio into MSM individually supplemented with 500 mg L^−1^ of typical PAEs pollutants (DBP and DMP) and the major intermediate metabolites (PA, catechol (CAT) and PCA). The cultures were incubated at 28 °C and 200 rpm for 7 days. The residual concentrations of each substrate were quantified by HPLC as described above.

### 2.5. Whole-Genome Sequencing of the Representative Efficient DBP-Degrading Bacteria

Whole-genome sequencing of the representative efficient DBP-degrading bacteria was performed by Majorbio Bioinformatics Technology Co., Ltd. (Shanghai, China). Coding sequences (CDS) in the genome were predicted using Prodigal v2.6.3 [27], while plasmid genes were predicted using GeneMarkS v4.3 [28]. Functional annotation of the predicted CDS was carried out using sequence alignment tools, including BLASTP v2.13.0, Diamond v2.1.8, and HMMER v3.3.2, based on databases such as NR, Swiss-Prot, Pfam (Protein families database), Gene Ontology (GO), Clusters of Orthologous Groups of Proteins (COG), Kyoto Encyclopedia of Genes and Genomes (KEGG), and Carbohydrate-Active Enzymes database (CAZY).

### 2.6. Data Analysis

One-way analysis of variance (ANOVA) was performed using SPSS v27.0 followed by a least significant difference (LSD) test to analyze significant differences in microbial growth and degradation efficiency of PAEs and their intermediates. Prior to analysis, the data were tested for normality and homogeneity of variance. The statistical significance level was set at *p* = 0.05. All experiments were conducted with three biological replicates.

## 3. Results and Discussion

### 3.1. Microbial Community Response to High Concentration of DMP/DBP Stress in Recycled Plastic Wastewater Treatment Plant

#### 3.1.1. Microbial Community Structure Response to High Concentration of DMP/DBP Stress in Recycled Plastic Wastewater Treatment Plant

**Figure 1:**
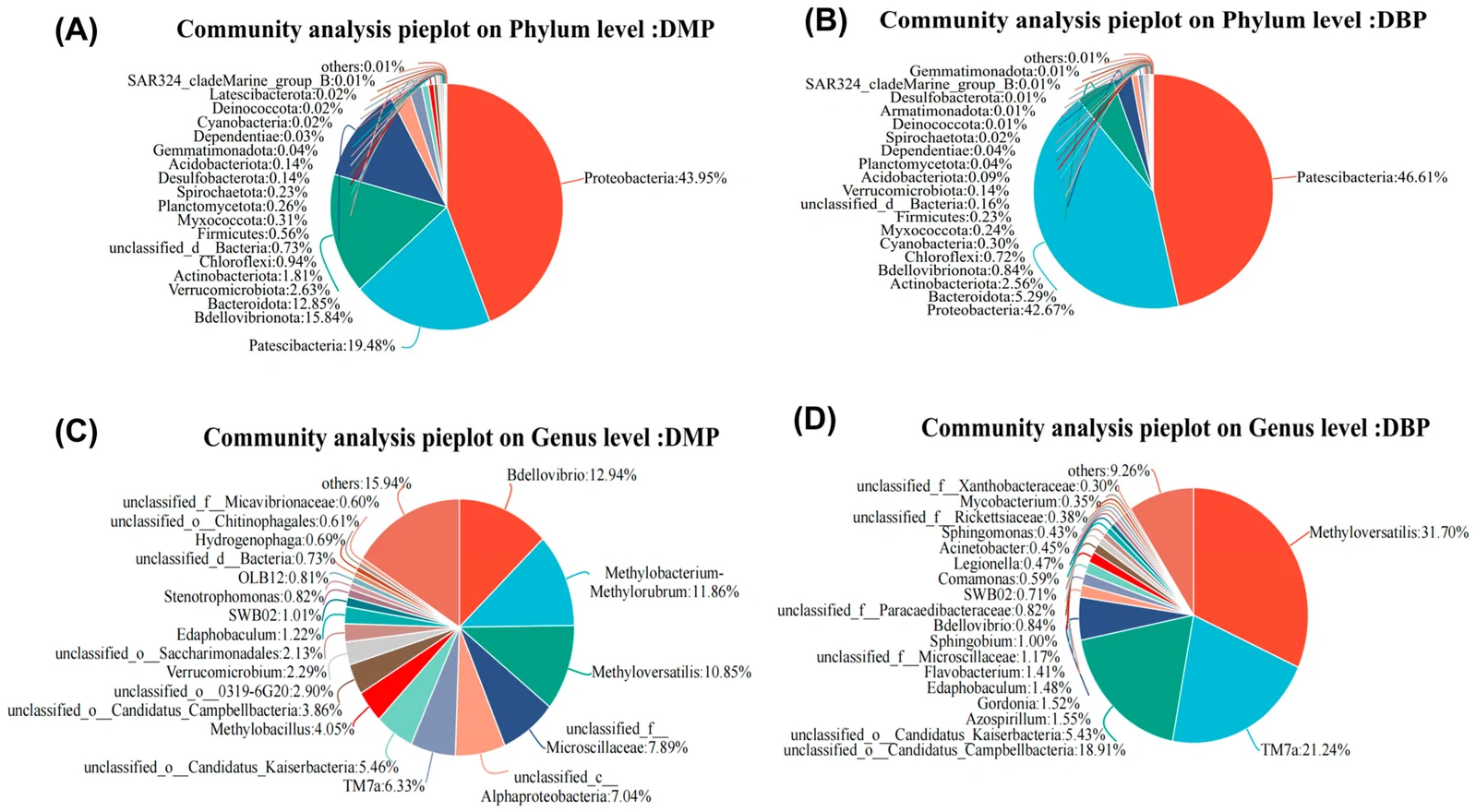
Microbial community structure under DMP/DBP stress in recycled plastic sewage treatment plant. (**A**,**B**) Relative abundance at phylum level under DMP and DBP stress. (**C**,**D**) Relative abundance at genus level under DMP and DBP stress.

High-throughput sequencing results demonstrated that the Shannon–Wiener indices were 4.11 and 2.86 under 500 mg L^−1^ DMP and 500 mg L^−1^ DBP stress, respectively (Table 1). Under DMP stress, 22 phyla and 278 genera were detected, and the dominant phyla were *Proteobacteria* (43.95%), *Patescibacteria* (19.48%), and *Bdellovibrionota* (15.84%). The dominant genera were *Bdellovibrio* (12.94%), *Methylobacterium* (11.86%), and *Methyloversatilis* (10.85%) (Table 1; Figure 1). Under DBP stress, 23 phyla and 250 genera were detected and the dominant phyla were *Proteobacteria* (42.67%), *Patescibacteria* (46.61%), and *Bacteroidota* (5.29%). The dominant genera were *Methyloversatilis* (31.70%), *TM7a* (21.24%), *Azospirillum* (1.55%), and *Gordonia* (1.52%). It was reported that many members of *Methylobacterium* [29], *Patescibacteria* [30], *Proteobacteria* [23], *Gordonia* [1], and *Bacteroidota* [31,32] could efficiently degrade PAEs.

Although the diversity of the microbial community decreased in the recycled plastic wastewater treatment plant, PAEs-degrading bacteria were predominant under DMP/DBP stress compared with those in domestic wastewater treatment plants [33] (Table 1; Figure 1). This might be attributed to directional selection pressure [34] and interspecific competitive exclusion effects [35].

#### 3.1.2. DMP/DBP-Degrading Performance of Microbial Community

**Figure 2:**
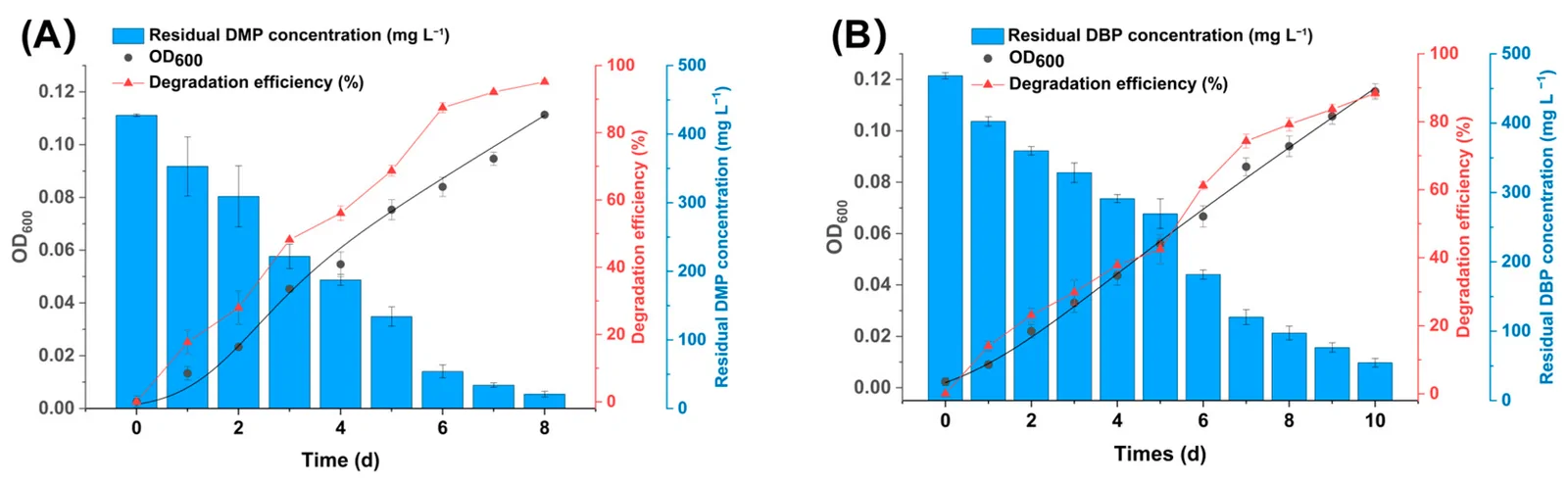
Microbial growth and degradation performance of DMP (**A**) and DBP (**B**) by microbial community in recycled plastic sewage treatment plant.

The study demonstrated that the degradation rate of DMP (95.13%) by the microbial community in the recycled plastic wastewater treatment plant was higher than that of DBP (88.35%) at a concentration of 500 mg L^−1^ (Figure 2). Compared with DBP, the shorter alkyl side chain of DMP facilitated its access to the active site of hydrolytic enzymes [36,37]. Additionally, the higher water solubility of DMP ensured a greater aqueous concentration and better bioavailability to microorganisms [38]. From a metabolic pathway perspective, DMP occupied a downstream position in the PAEs degradation pathway and its simple molecular structure made it easier to be mineralized by microbial communities [39].

### 3.2. Screening and Identification of DMP/DBP-Degrading Bacteria

Further study revealed that abundant culturable DMP/DBP-degrading bacteria existed in the recycled plastic sewage treatment plant. A total of 68 strains with DMP-degrading capability were isolated from solid MSM supplemented with 500 mg L^−1^ DMP, belonging to three phyla, 19 genera and 27 species (Table S2). Among them, the dominant bacterial phyla were Proteobacteria (41 strains) and Firmicutes (25 strains), while the predominant genera included *Bacillus* (16 strains), *Raoultella* (12 strains), *Acinetobacter* (five strains), *Hyphomicrobium* (five strains), and *Staphylococcus* (five strains) (Table S2).

A total of 51 DBP-degrading strains were isolated, belonging to three phyla, 11 genera, and 20 species (Table S3). Among them, the dominant phyla were Proteobacteria (27 strains) and Firmicutes (22 strains), while the predominant genera included *Bacillus* (22 strains), *Brucella* (seven strains), *Agrobacterium* (four strains), and *Paenirhizobium* (four strains) (Table S3).

Notably, this study screened numerous PAEs-degrading bacteria rarely reported from recycled plastic wastewater treatment plants, including 11 genera such as *Paenirhodobacter*, *Ciceribacter*, *Neorhizobium*, *Raoultella*, *Hyphomicrobium*, *Methylorubrum*, *Ancylobacter*, *Niallia*, *Priestia*, *Fictibacillus* and one potential new species SWLYDMP 33. The similarity of the 16S rRNA gene sequence between SWLYDMP 33 and the *Hyphomicrobium facile* strain was 98.58%, which was lower than the baseline threshold of 98.65%, suggesting that it was a potential new species [39]. However, this definitive claim needs further studies such as Average Nucleotide Identity or digital DNA-DNA hybridization in the future. Among them, *Niallia* [40], *Priestia* [41], and *Fictibacillus* [42] were known for their high resistance to environmental stress. *Hyphomicrobium* [43], *Methylorubrum* [44], and *Ancylobacter* [45] were typical methylotrophic or facultative C_1_ metabolic bacteria capable of rapidly consuming short-chain alcohols generated during PAEs degradation. Thus, the rare PAEs-degrading genera and potential new species isolated in this study not only substantially expanded the diversity of PAEs-degrading microorganisms but also offered unique potential advantages for practical in situ remediation in complex environments.

The study also revealed that although the phylum *Proteobacteria* was dominant among the DMP/DBP-degrading bacteria obtained via high-throughput sequencing and the cultivation-dependent approach, the screened dominant DMP/DBP-degrading bacterial genera such as *Bacillus* and *Brucella* were discrepant with those dominant genera like *Methyloversatilis* and *TM7a* in high-throughput sequencing. This discrepancy might be attributed to methodological biases and the decoupling of taxonomic abundance and functional activity [46]. For example, high-throughput sequencing captured the total genetic signature of the entire community, including both dormant and unculturable cells [46]. In contrast, the screened DMP/DBP-degrading bacteria on selective medium were typically culturable r-strategists capable of utilizing DMP/DBP as the sole carbon source [47]. Therefore, the combination of the two approaches above provided complementary insights into both community structure and culturable functional resources involved in PAEs degradation.

### 3.3. DMP/DBP-Degrading Capacity of the Screened Bacteria

The study revealed that the recycled plastic wastewater treatment plant harbored abundant efficient DMP/DBP-degrading bacteria. The degradation efficiencies of the 68 DMP-degrading strains ranged from 10.20% to 90.38%, with 16 strains (23.53%) exhibiting efficiencies exceeding 75% (Table S2). Strain SWLYDMP 24 displayed the highest degradation capacity at 90.38%, surpassing all other isolated strains and exceeding that of *Acinetobacter baumannii* DP-2 isolated from activated sludge of sewage treatment plant (42.5%) [48].

The degradation rates of the 51 DBP-degrading strains ranged from 10.09% to 99.90%, with 19 strains (37.25%) achieving efficiencies above 75% (Table S3). Among them, SWLYDBP 51 exhibited the highest degradation efficiency (99.90%) (Table S3), surpassing all other isolates in this study as well as previously reported strains such as *Gordonia* sp. from river sediment (78–98%), *Providencia* sp. 2D from compost (89.0%) [49,50], and the strain *Gordonia* sp. YC-JH1 from petroleum-contaminated soil (4–14%) [51]. Furthermore, based on high-throughput sequencing, SWLYDBP 51 belonged to the dominant genus *Gordonia* and might play a key role in PAEs-degrading in the recycled plastic wastewater treatment plant. Therefore, SWLYDBP 51 was selected as the representative efficient DBP-degrading strain for further study.

### 3.4. The Representative Efficient DBP-Degrading Strain SWLYDBP 51 Could Effectively Degrade Various Typical PAEs and Their Metabolic Intermediates

**Figure 3:**
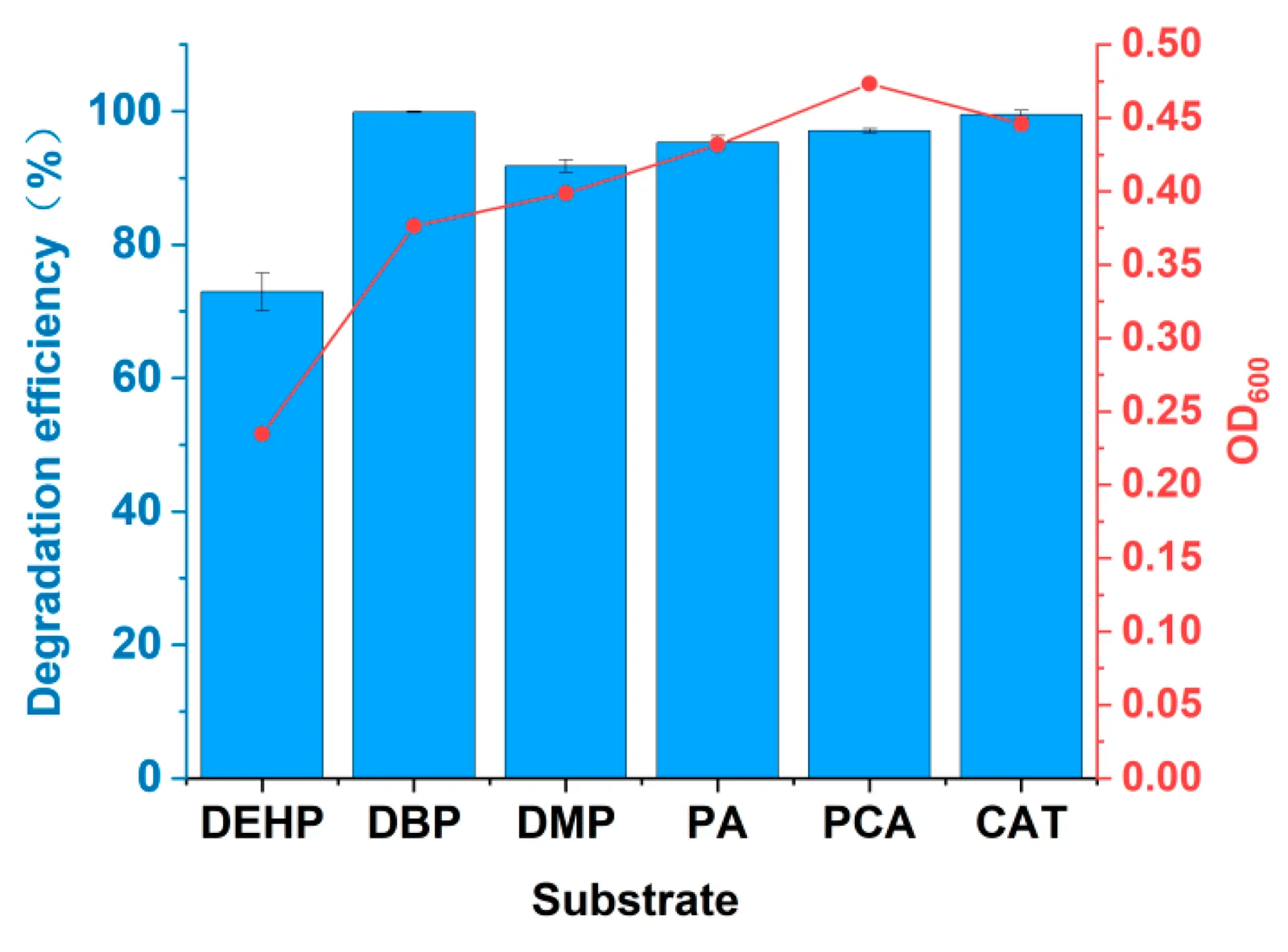
Microbial biomass and substrate profile of strain SWLYDBP 51.

The study demonstrated that SWLYDBP strain 51 could not only efficiently degrade typical PAEs, including DMP, DBP and di(2-ethylhexyl) phthalate (DEHP), but also could effectively degrade typical intermediate metabolites, such as PA, PCA, and CAT, indicating its broad substrate profile (Figure 3). Its degradation efficiencies for DEHP, DBP, DMP, PA, PCA, and CAT were 72.94%, 99.90%, 91.79%, 95.36%, 97.07%, and 99.50%, respectively.

### 3.5. Genomic Annotation of the Representative Efficient DBP-Degrading Strain SWLYDBP 51

**Figure 4:**
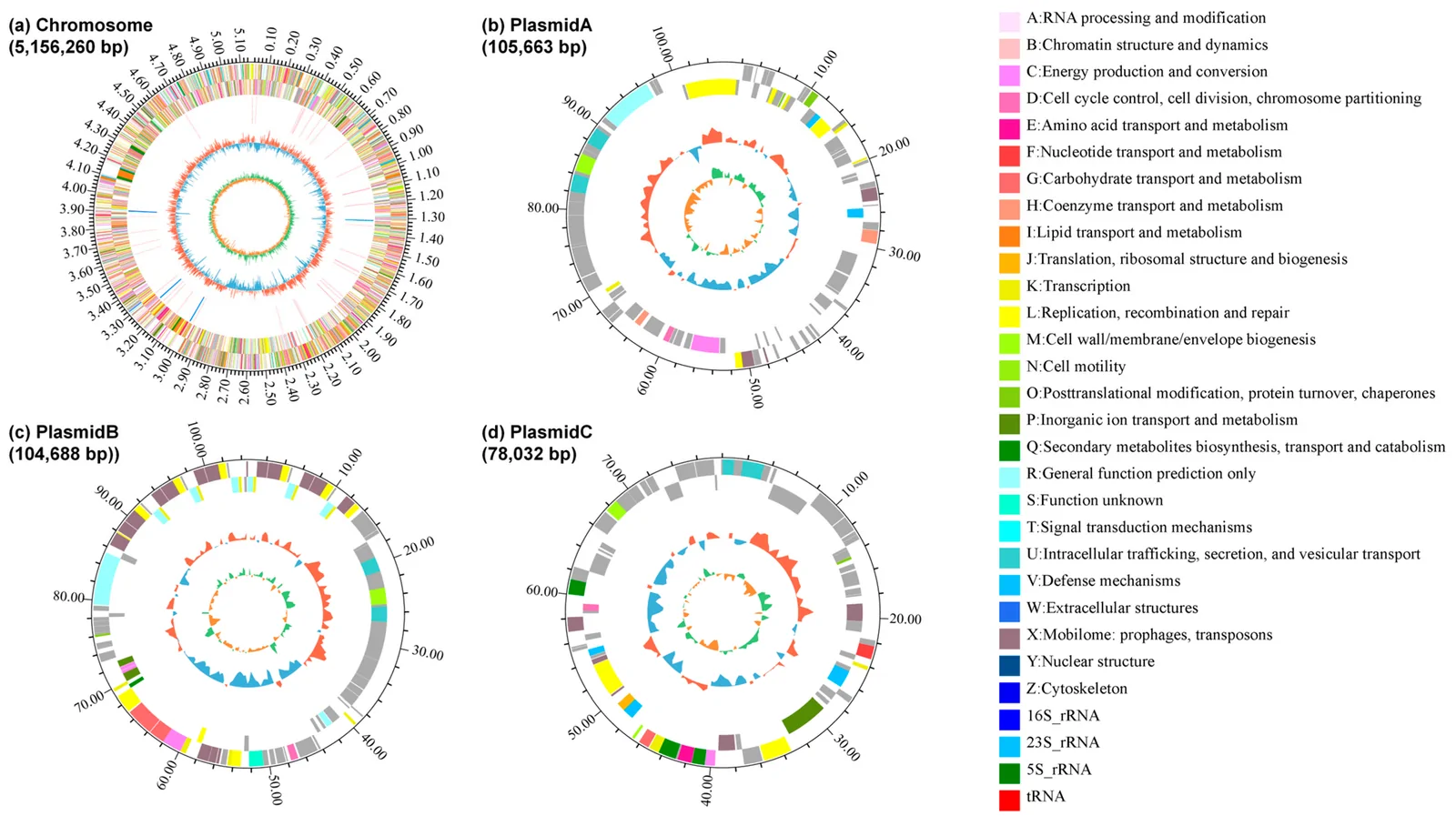
Circular genome map of strain SWLYDBP 51.

Given the SWLYDBP 51 affiliation with a dominant genus and superior broad-spectrum degradation performance, whole-genome sequencing for SWLYDBP 51 was conducted and revealed that its genome comprised one chromosome (5,156,260 bp) and three plasmids (Figure 4). The functional annotation statistics for the coding sequences (CDS) of SWLYDBP 51 against major public databases (NR, Swiss-Prot, Pfam, COG, GO, and KEGG) are presented in Table 2.

**Figure 5:**
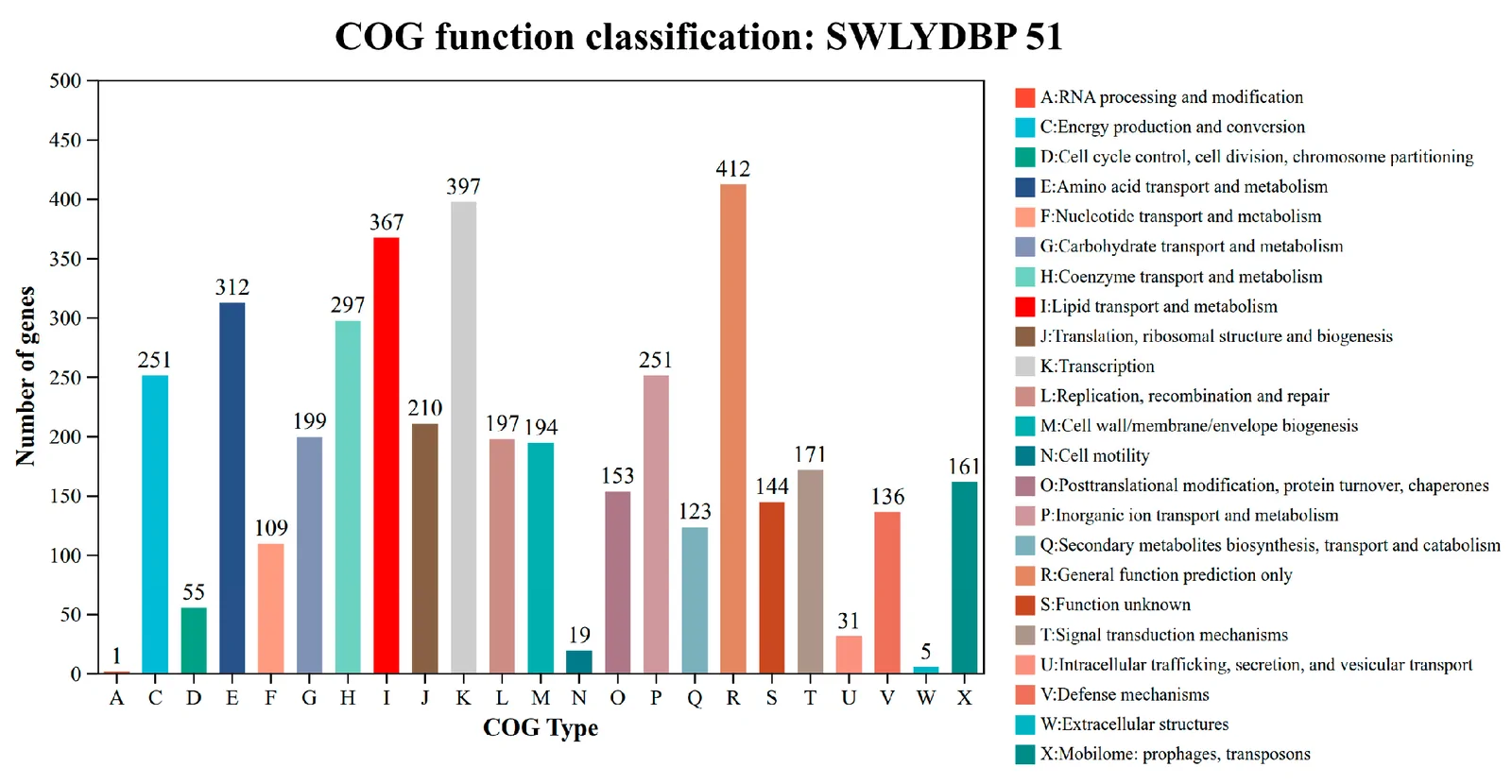
Statistical chart of COG annotation classification for strain SWLYDBP 51.

A total of 3793 genes (76.35%) in the SWLYDBP 51 genome were successfully annotated into 23 functional categories in the COG database (Figure 5). A large proportion of these genes were assigned into functional groups closely associated with cellular metabolism, including transcription (K), amino acid transport and metabolism (E), coenzyme transport and metabolism (H), energy production and conversion (C), and carbohydrate transport and metabolism (G) (Figure 5). This indicated that SWLYDBP 51 strain harbored robust metabolic potential, active transcriptional regulation, and efficient maintenance of cellular functions.

**Figure 6:**
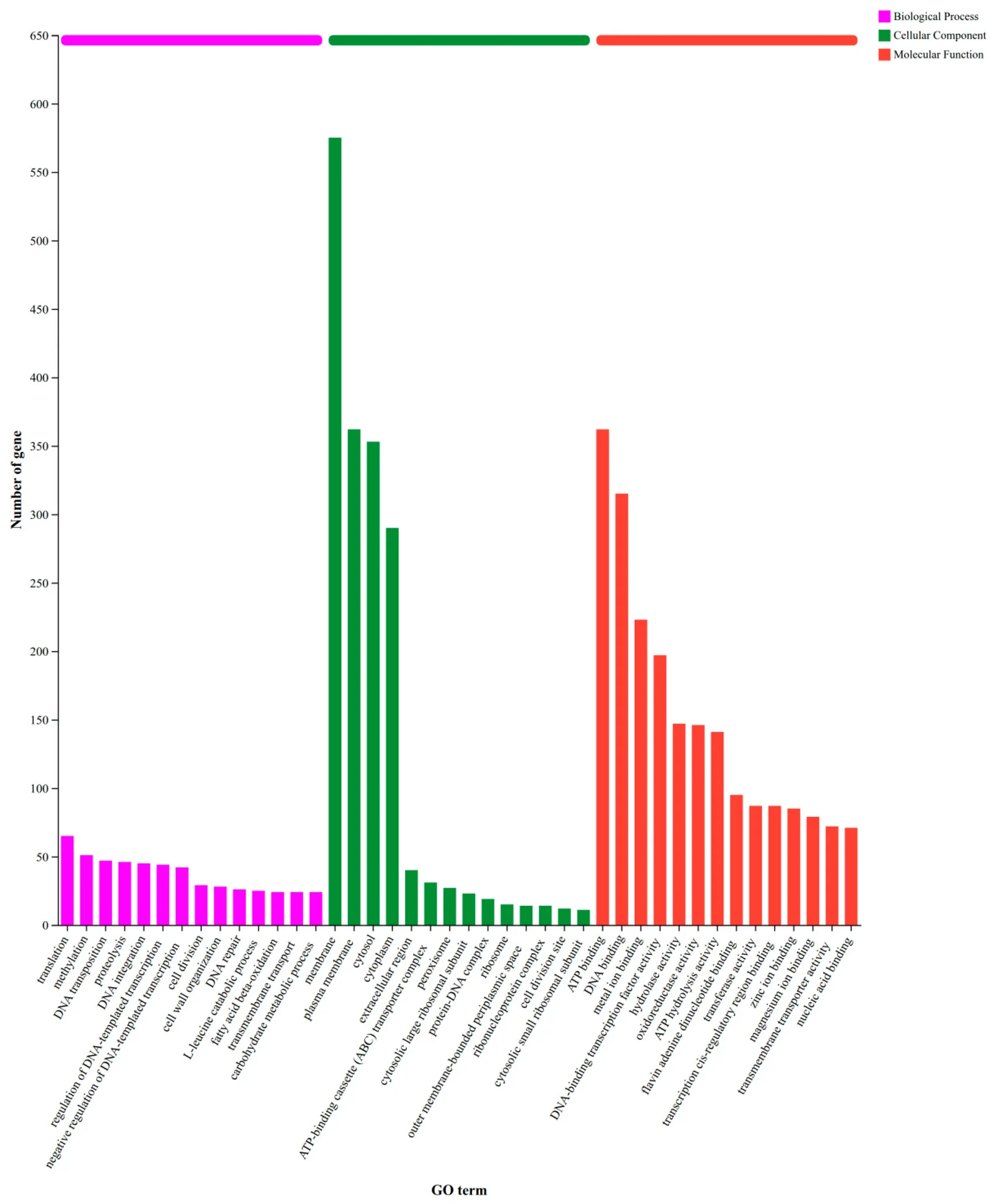
Statistical chart of GO annotation classification for strain SWLYDBP 51.

A total of 3399 genes (68.42%) were annotated against the GO database. Genes related to molecular function accounted for the highest proportion at 84.88% (2885 genes), followed by those related to cellular components and biological processes (Figure 6).

**Figure 7:**
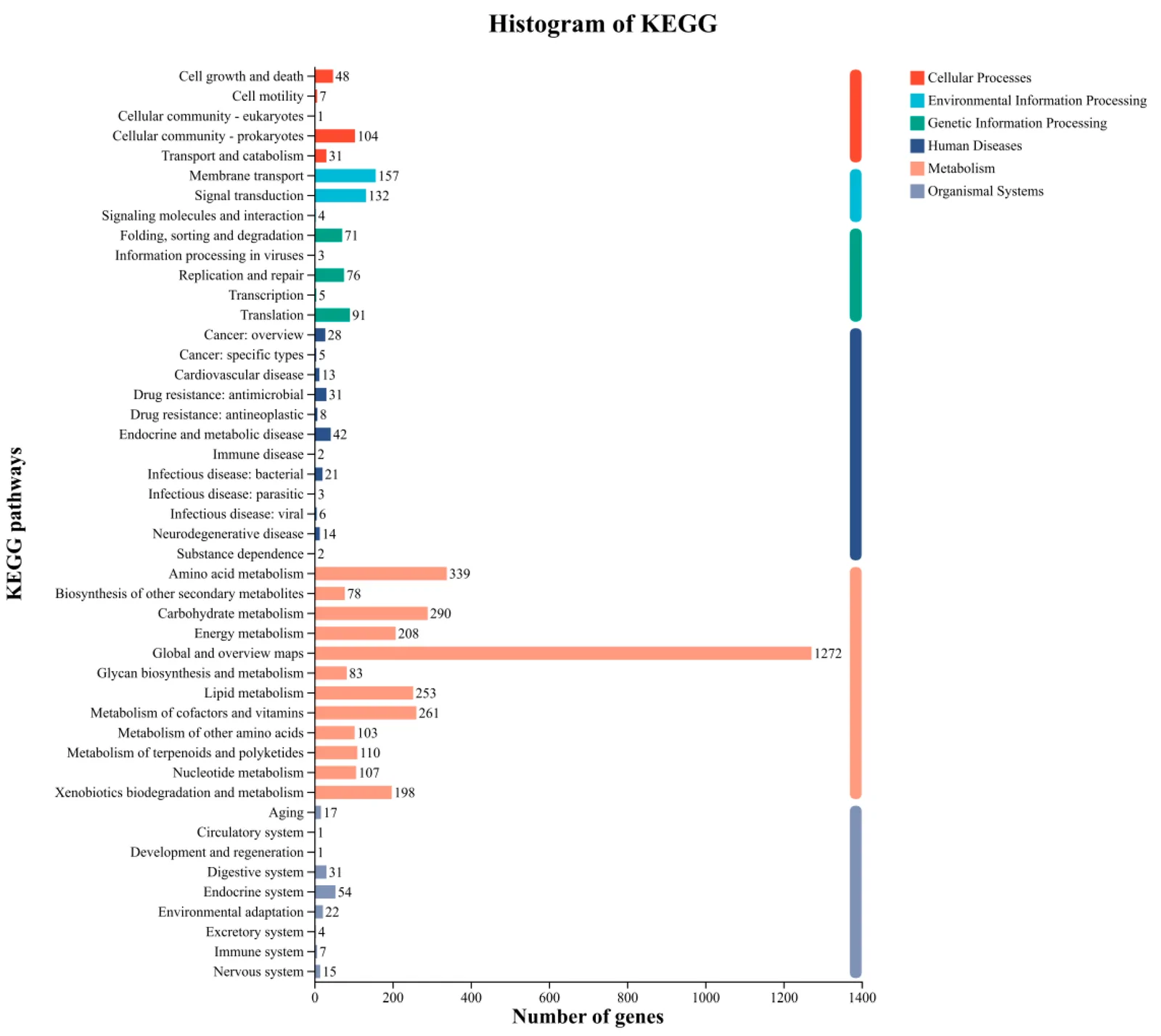
Statistical chart of KEGG annotation classification for strain SWLYDBP 51.

Analysis of KEGG annotations revealed that most genes (3302 genes) of SWLYDBP 51 strain genome were related to metabolic pathways, followed by environmental information processing (293 genes), genetic information processing (246 genes), and cellular processes (191 genes) (Figure 7).

Notably, 198 genes of strain SWLYDBP 51 were annotated as involved in xenobiotic biodegradation and the metabolic pathway, indicating its potential to effectively metabolize xenobiotic organic pollutants. Furthermore, the genes related to membrane transport (157 genes) and signal transduction (132 genes) were dominant in environmental information processing pathways. The genes related to translation (91 genes) and replication/repair (76 genes) were dominant in genetic information processing. These annotations suggested that these genes played critical roles in enhancing protein transport and secretion, thereby enabling SWLYDBP 51 to efficiently degrade exogenous pollutants and effectively resist the effects of harmful pollutants.

**Figure 8:**
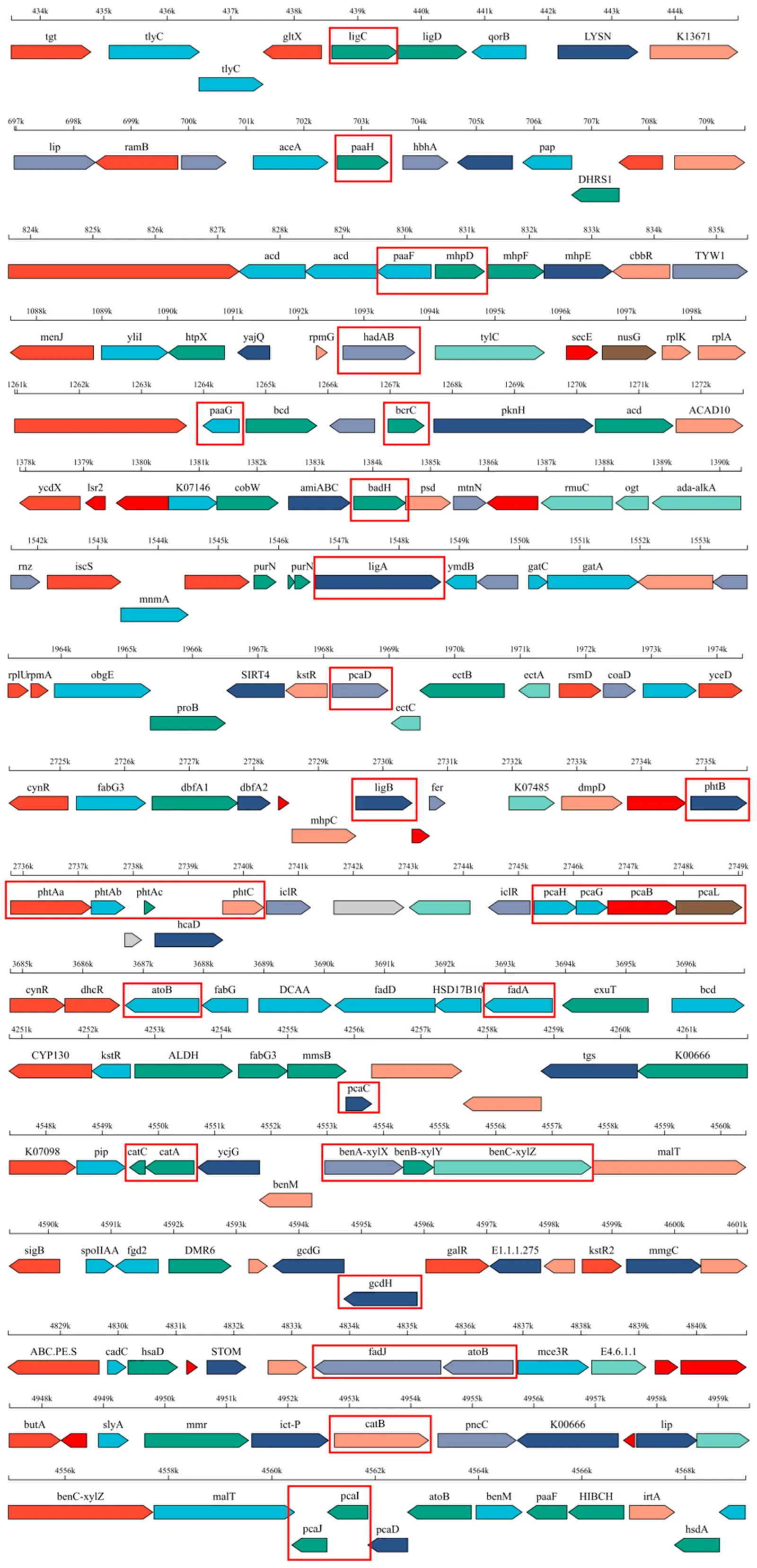
The putative key genes responsible for the degradation of PA and its metabolites in strain SWLYDBP 51 based on genomic annotations. Note: The length and colors of the arrows represent the gene length and COG functional types, respectively.

This study revealed that the genome of SWLYDBP 51 strain harbored abundant PAEs-degrading genes. In total, 95 potential esterase/hydrolase genes and 77 PA-degrading genes were annotated, 84% of which exhibited 100% sequence homology. Significantly, compared with other previously reported *Gordonia* strains, such as *Gordonia* sp. RL-BY03 [52], *Gordonia* sp. YC-JH1 [51], *Gordonia* sp. LUNF6 [20], and *Gordonia alkanivorans* GH-1 [1], SWLYDBP 51 strain not only harbored classical PA-degrading gene clusters such as *pht* and *pca* clusters, but also contained *ben*, *cat*, *lig*, *fad*, *paa* and *bad* clusters, along with genes such as *mhpD* and *atoB*. Among these, *lig*, *fad,* and *bad* clusters and *mhpD* gene were scarcely reported in *Gordonia* genera (Tables S4 and S5; Figure 8). The abundant PAEs degradation functional genes in strain SWLYDBP 51 provided a solid genetic foundation for its good performance of PAEs degradation, although the actual functional roles of these genes required further experimental validation.

### 3.6. Putative Complicated DBP-Degrading Pathways for Strain SWLYDBP 51 Based on Genomic Annotation

**Figure 9:**
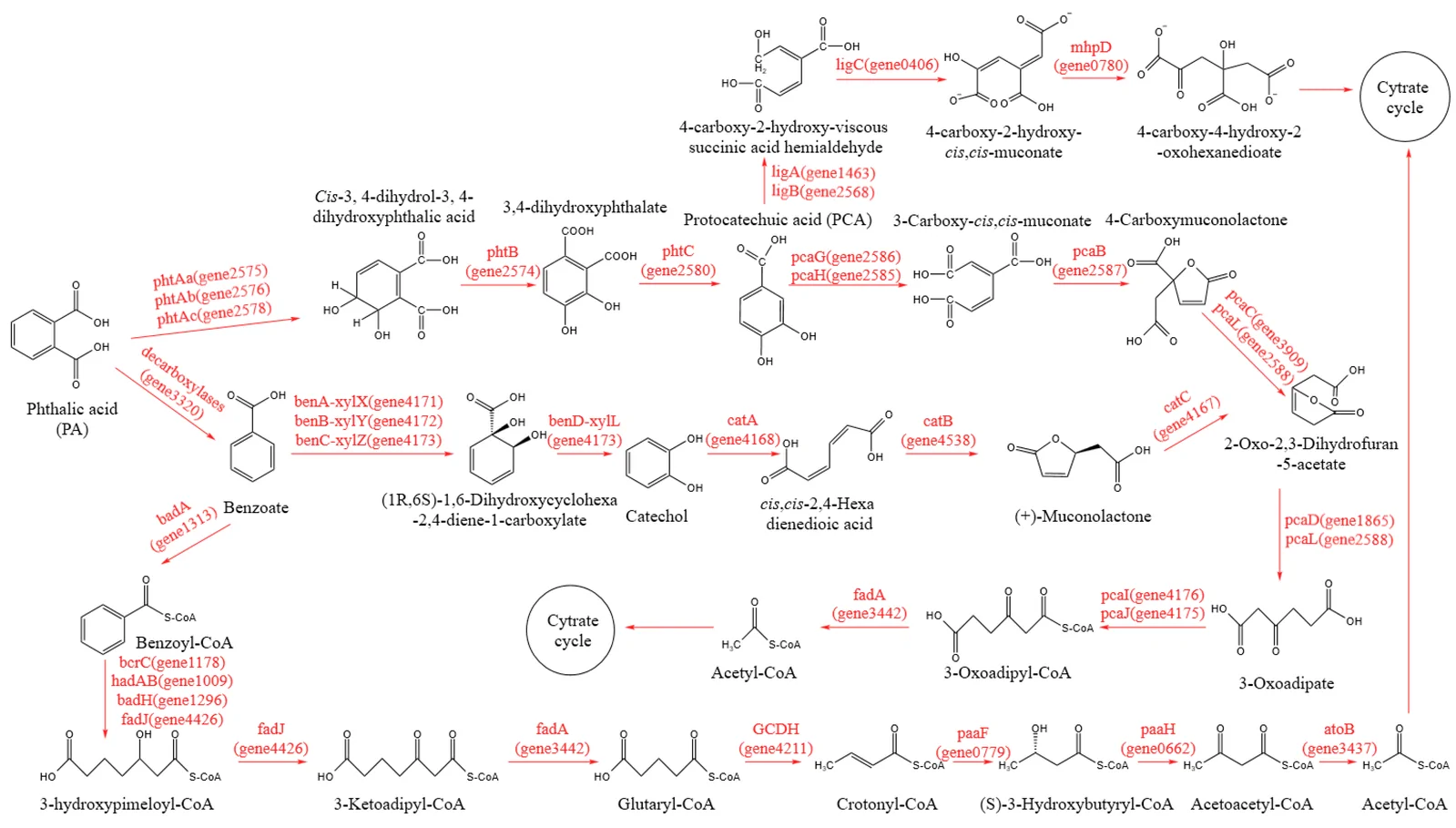
Putative degrading pathways of PA and its metabolites by strain SWLYDBP 51 based on genomic annotations.

Compared with other *Gordonia* strains annotated to possess relatively simple PA-degrading pathways or only genes involved in PA dioxygenation or PCA ring-opening [1,15,52], strain SWLYDBP 51 was predicted to harbor a complex DBP metabolic network based on its abundant PAEs-degrading genes repertoire revealed by genomic analysis (Figure 9). The most possible main pathway was that type I esterase (gene 0858) and type II esterase (gene 2572) hydrolyzed DBP into PA. In addition, genomic evidence suggested that strain SWLYDBP 51 might degrade DBP to PA via type III esterase. PA was subsequently inferred to be metabolized via the *pca* gene cluster to PCA, and then converted into 3-oxoadipyl-CoA by the *pht* gene cluster. Then, 3-oxoadipyl-CoA would be metabolized into acetyl-CoA by thiolase *fadA* (gene 3442) and enter the TCA cycle for complete mineralization (Figure 9).

Moreover, PA might also be converted to benzoic acid by decarboxylase (gene 3320). Subsequently, benzoic acid was speculated to be degraded to β-ketohexanediol lactone via the *ben* gene cluster, dehydrogenase *benD-xylL* (gene 4173), and the *cat* gene cluster, then entering the main metabolic pathway above. Alternatively, benzoic acid might be further transformed into acetyl-CoA prior to entering the TCA cycle via such genes as *badA* (gene 1313), *bcrC* (gene 1178), *hadAB* (gene 1009), *badH* (gene 1296), and *thiolase atoB* (gene 3437) (Figure 9). The actual involvement of these genes in PAEs degradation and the precise PAEs degradation pathway needed to be validated by gene expression analysis, enzyme activity assays, intermediate metabolite identification, proteomic evidence, or functional genetic experiments. Nevertheless, the putative substantial PAEs-degrading gene redundancy and complex PAEs degradation network inferred from the genomic analysis of SWLYDBP 51 provided a valuable foundation for future mechanistic studies and highlighted its genetic potential for PAEs bioremediation under complex environmental conditions.

## 4. Conclusions

This study demonstrated that the recycled plastic wastewater treatment plant harbored abundant efficient DMP/DBP-degrading bacteria. High-throughput sequencing revealed 22 phyla and 278 genera resistant to 500 mg L^−1^ DMP and 23 phyla and 250 genera resistant to 500 mg L^−1^ DBP. Further study demonstrated that 19 genera and 27 species of DMP-degrading bacteria, along with 11 genera and 20 species of DBP-degrading bacteria, were successfully isolated, including a potential novel species and 11 genera seldom reported with the function of PAEs degradation. DBP-degrading strain SWLYDBP 51 was selected for whole-genome sequencing based on its affiliation with the dominant genus *Gordonia*, and its strong degradation performance for both PAEs and their intermediates (PA, PCA, and CAT). Abundant PAEs-degrading functional genes and a complex degradation network were predicted based on genomic annotation, suggesting that SWLYDBP 51 held promising genetic potential for effective DBP bioremediation under complex environmental conditions. However, experimental validation was required to confirm the actual catabolic pathway and the functional roles of individual genes. Overall, this study substantially enriched the reservoir of efficient PAEs-degrading microbial resources.

## Figures and Tables

**Table 1:** Microbial community diversity under high concentration of DMP/DBP in recycled plastic sewage treatment plant.

DMP/DBP Stress	Chao1	Shannon	Simpson	Phyla	Genus
DMP	692	4.11	0.04	22	278
DBP	636	2.86	0.16	23	250

**Table 2:** Functional annotation statistics for CDS of SWLYDBP 51 in major public databases.

Database	NR	Swiss-Prot	Pfam	COG	GO	KEGG
Quantity	4935	3405	4030	3793	3399	3409

## Data Availability

The raw data supporting the conclusions of this article will be made available by the authors on request.

## Supplementary Materials

The following supporting information can be downloaded at: https://www.mdpi.com/article/10.3390/microorganisms14091896/s1. Table S1. DMP/DBP concentration and degradation rate in inlet, outflow and activated sludge of recycled plastic wastewater treatment plant. Table S2. Online BLAST comparison results of 16S rRNA gene sequences and DMP degradation of DMP-degrading bacteria. Table S3. Online BLAST comparison results of 16S rRNA gene sequences and degradation rate of DBP-degrading bacteria. Table S4. Genes encoding esterase/hydrolase in SWLYDBP 51 strain genome. Table S5. Annotation of genes degrading PA and its intermediate metabolites in SWLYDBP strain 51 genome.
