# Supplementary material for: Rich Efficient Short-Chain Phthalate Esters-Degrading Bacteria and Their Degradation Mechanisms in Recycled Plastic Wastewater Treatment Plant
Source: Microorganisms. 2026 Aug 26;14(9):1896. doi: 10.3390/microorganisms14091896 (PMC13609268; doi:10.3390/microorganisms14091896)
Supplement: Supplementary file 1 [file microorganisms-14-01896-s001.zip › microorganisms-4471980-supplementary.pdf]

Table S1 DMP/DBP concentration and degradation rate in inlet, outflow and activated sludge of recycled plastic wastewater treatment plant.

PAEs kind	Inlet concentration (mg L^{-1})	Outflow concentration (mg L^{-1})	Sludge concentration(mg g^{-1})	Degradation rate (%)
DMP	203.17 ± 7.44	97.06 ± 5.52	43.73 ± 1.18	52.23 ± 2.05
DBP	86.99 ± 3.29	75.58 ± 1.85	116.66 ± 5.90	13.12 ± 0.59

Note: The data were expressed as mean ± standard deviation (n = 3).

Table S2 Online BLAST comparison results of 16S rRNA gene sequences and DMP degradation of DMP-degrading bacteria.

Strain number	DMP degradation rate (%)	Gene similarity (%)	Species classification
SWLYDMP 1	55.83 ± 1.56 i	99.28	*Raoultella ornithinolytica*
SWLYDMP 2	80.32 ± 3.16 c	99.71	*Raoultella ornithinolytica*
SWLYDMP 3	74.65 ± 0.29 de	99.27	*Raoultella ornithinolytica*
SWLYDMP 4	74.27 ± 0.65 de	99.16	*Raoultella ornithinolytica*
SWLYDMP 5	76.60 ± 1.65 d	99.58	*Raoultella ornithinolytica*
SWLYDMP 6	67.47 ± 4.00 f	99.51	*Raoultella ornithinolytica*
SWLYDMP 7	62.08 ± 0.36 g	99.42	*Raoultella ornithinolytica*
SWLYDMP 8	78.48 ± 0.63 cd	99.51	*Raoultella planticola*
SWLYDMP 9	86.90 ± 0.33 ab	99.51	*Raoultella planticola*
SWLYDMP 10	73.44 ± 1.15 e	99.44	*Raoultella planticola*
SWLYDMP 11	80.62 ± 5.49 c	99.09	*Raoultella planticola*
SWLYDMP 12	72.78 ± 0.19 e	99.03	*Raoultella planticola*
SWLYDMP 13	78.91 ± 0.19 cd	99.20	*Escherichia fergusonii*
SWLYDMP 14	6.40 ± 1.43 xy	99.07	*Escherichia fergusonii*
SWLYDMP 15	22.92 ± 0.26 t	99.58	*Acinetobacter radioresistens*
SWLYDMP 16	32.64 ± 0.71 r	99.37	*Acinetobacter radioresistens*
SWLYDMP 17	68.55 ± 0.51 ef	99.61	*Acinetobacter radioresistens*
SWLYDMP 18	34.91 ± 1.48 pqr	99.21	*Acinetobacter radioresistens*
SWLYDMP 19	75.06 ± 0.55 de	99.58	*Acinetobacter radioresistens*
SWLYDMP 20	67.78 ± 0.44 f	99.55	*Paenirhodobacter ferrireducens*
SWLYDMP 21	79.47 ± 0.68 cd	99.85	*Ciceribacter naphthalenivorans*
SWLYDMP 22	35.14 ± 1.60 pqr	99.78	*Ciceribacter naphthalenivorans*
SWLYDMP 23	59.35 ± 0.66 gh	99.64	*Agrobacterium pusense*
SWLYDMP 24	90.38 ± 0.05 a	99.56	*Agrobacterium pusense*
SWLYDMP 25	46.53 ± 4.20 k	98.87	*Rhizobium arsenicireducens*
SWLYDMP 26	74.68 ± 0.82 de	99.26	*Rhizobium arsenicireducens*
SWLYDMP 27	84.24 ± 0.27 b	99.20	*Neorhizobium petrolearium*
SWLYDMP 28	88.48 ± 0.26 a	99.78	*Neorhizobium petrolearium*

Continued Table S2 Online BLAST comparison results of 16S rRNA gene sequences and DMP degradation of DMP-degrading bacteria.

Strain number	DMP degradation rate (%)	Gene similarity (%)	Species classification
SWLYDMP 30	8.24 ± 2.38 wx	98.89	*Hyphomicrobium facile*
SWLYDMP 31	10.20 ± 0.92 w	98.75	*Hyphomicrobium facile*
SWLYDMP 32	46.76 ± 0.98 k	98.96	*Hyphomicrobium facile*
SWLYDMP 33	4.92 ± 0.56 y	98.58	*Hyphomicrobium facile*
SWLYDMP 34	77.60 ± 0.91 cd	99.34	*Methylorubrum populi*
SWLYDMP 35	35.92 ± 0.52 pqr	98.75	*Ancylobacter novellus*
SWLYDMP 36	51.56 ± 1.10 j	99.85	*Brucella lupini*
SWLYDMP 37	67.45 ± 0.70 f	99.78	*Brucella lupini*
SWLYDMP 38	79.48 ± 0.30 cd	99.04	*Brucella lupini*
SWLYDMP 39	5.65 ± 0.67 xy	99.85	*Brucella lupini*
SWLYDMP 40	80.66 ± 0.70 c	98.67	*Comamonas testosteroni*
SWLYDMP 41	46.15 ± 0.39 kl	98.81	*Comamonas testosteroni*
SWLYDMP 42	46.03 ± 0.99 kl	99.57	*Bacillus paramycoides*
SWLYDMP 43	36.40 ± 0.83 pq	99.43	*Bacillus paramycoides*
SWLYDMP 44	13.50 ± 0.60 v	99.79	*Bacillus paramycoides*
SWLYDMP 45	5.60 ± 1.02 xy	99.92	*Bacillus paramycoides*
SWLYDMP 46	26.93 ± 0.57 s	99.86	*Bacillus paramycoides*
SWLYDMP 47	69.15 ± 3.57 ef	99.72	*Bacillus paramycoides*
SWLYDMP 48	40.90 ± 1.64 no	99.36	*Bacillus paramycoides*
SWLYDMP 49	72.43 ± 1.59 e	99.86	*Bacillus paramycoides*
SWLYDMP 50	57.04 ± 1.05 hi	99.93	*Bacillus paramycoides*
SWLYDMP 51	17.22 ± 1.32 u	99.54	*Bacillus paramycoides*
SWLYDMP 52	23.77 ± 1.05 t	99.63	*Bacillus paramycoides*
SWLYDMP 53	46.23 ± 0.90 kl	99.72	*Bacillus paramycoides*
SWLYDMP 54	45.68 ± 0.89 klm	99.86	*Bacillus toyonensis*
SWLYDMP 55	71.62 ± 2.07 e	99.65	*Bacillus toyonensis*
SWLYDMP 56	34.85 ± 0.64 pqr	99.50	*Bacillus pacificus*
SWLYDMP 57	37.93 ± 1.12 op	99.93	*Bacillus aerius*
SWLYDMP 58	43.12 ± 0.33 lmn	99.01	*Niallia nealsonii*

Continued Table S2 Online BLAST comparison results of 16S rRNA gene sequences and DMP degradation of DMP-degrading bacteria.

Strain number	DMP degradation rate (%)	Gene similarity (%)	Species Classification
SWLYDMP 59	33.91 ± 0.23 qr	99.16	*Niallia nealsonii*
SWLYDMP 60	73.17 ± 4.74 e	99.72	*Priestia flexa*
SWLYDMP 61	61.76 ± 2.07 g	98.94	*Fictibacillus phosphorivorans*
SWLYDMP 62	42.64 ± 0.50 mn	99.71	*Staphylococcus epidermidis*
SWLYDMP 63	13.58 ± 0.78 v	99.86	*Staphylococcus edaphicus*
SWLYDMP 64	44.89 ± 0.91 klm	99.72	*Staphylococcus edaphicus*
SWLYDMP 65	67.77 ± 1.04 f	99.65	*Staphylococcus capitis*
SWLYDMP 66	82.47 ± 3.39 bc	99.55	*Staphylococcus equorum*
SWLYDMP 67	34.32 ± 0.33 pqr	99.72	*Streptomyces rochei*
SWLYDMP 68	84.02 ± 0.16 b	98.95	*Streptomyces viridosporus*

Table S3 Online BLAST comparison results of 16S rRNA gene sequences and degradation rate of DBP-degrading bacteria

Strain number	DBP degradation rate (%)	Gene similarity (%)	Species Classification
SWLYDBP 1	39.29 ± 0.11 w	99.57	*Bacillus toyonensis*
SWLYDBP 2	63.62 ± 7.02 i	99.36	*Bacillus toyonensis*
SWLYDBP 3	65.29 ± 8.95 h	99.43	*Bacillus toyonensis*
SWLYDBP 4	55.05 ± 7.00 o	99.42	*Bacillus toyonensis*
SWLYDBP 5	82.18 ± 0.36 f	99.93	*Bacillus toyonensis*
SWLYDBP 6	98.77 ± 0.02 ab	99.71	*Bacillus toyonensis*
SWLYDBP 7	89.29 ± 0.38 c	100.00	*Bacillus thuringiensis*
SWLYDBP 8	95.44 ± 0.22 c	99.93	*Bacillus thuringiensis*
SWLYDBP 9	54.22 ± 7.48 p	99.64	*Bacillus thuringiensis*
SWLYDBP 10	60.83 ± 8.13 jk	99.78	*Bacillus paramycoides*
SWLYDBP 11	62.66 ± 1.96 i	99.49	*Bacillus paramycoides*
SWLYDBP 12	13.49 ± 2.30 x	99.79	*Bacillus paramycoides*
SWLYDBP 13	51.35 ± 1.25 q	99.43	*Bacillus paramycoides*
SWLYDBP 14	40.07 ± 3.70 w	99.86	*Bacillus paramycoides*
SWLYDBP 15	52.25 ± 5.81 q	99.71	*Bacillus paramycoides*
SWLYDBP 16	64.45 ± 7.56 h	99.29	*Bacillus mojavensis*
SWLYDBP 17	38.90 ± 0.59 w	99.54	*Bacillus mojavensis*
SWLYDBP 18	49.98 ± 6.25 r	99.43	*Bacillus pacificus*
SWLYDBP 19	44.44 ± 8.02 u	99.13	*Streptomyces sampsonii*
SWLYDBP 20	54.71 ± 7.55 o	99.24	*Sphingopyxis soli*
SWLYDBP 21	53.95 ± 6.46 p	99.86	*Sphingopyxis soli*
SWLYDBP 22	46.93 ± 1.14 t	99.14	*Paenirhizobium* [illegible]
SWLYDBP 23	57.26 ± 6.99 n	99.37	*Paenirhizobium* [illegible]
SWLYDBP 24	42.25 ± 0.48 v	99.13	*Paenirhizobium* [illegible]
SWLYDBP 25	98.33 ± 0.07 b	99.41	*Paenirhizobium* [illegible]
SWLYDBP 26	59.56 ± 6.37 l	99.33	*Agrobacterium salinitolerans*
SWLYDBP 27	87.09 ± 0.04 d	99.63	*Agrobacterium salinitolerans*

Continued Table S3 Online BLAST comparison results of 16S rRNA gene sequences and degradation rate of DBP-degrading bacteria

Strain number	DBP degradation rate (%)	Gene similarity (%)	Species Classification
SWLYDBP 28	84.11 ± 0.04 e	99.04	*Paenirhizobium daejeonense*
SWLYDBP 29	49.12 ± 0.03 s	98.90	*Paenirhizobium daejeonense*
SWLYDBP 30	58.62 ± 1.61 m	99.85	*Brevundimonas bullata*
SWLYDBP 31	61.60 ± 0.48 j	99.84	*Aeromonas media*
SWLYDBP 32	75.27 ± 0.03 f	98.85	*Aeromonas media*
SWLYDBP 33	55.02 ± 0.58 o	99.28	*Aeromonas media*
SWLYDBP 34	97.37 ± 0.03 b	99.56	*Bacillus wiedmannii*
SWLYDBP 35	86.70 ± 0.06 d	98.88	*Bacillus stercoris*
SWLYDBP 36	74.90 ± 0.31 f	99.71	*Bacillus tequilensis*
SWLYDBP 37	87.46 ± 0.30 d	99.71	*Sphingomonas sanguinis*
SWLYDBP 38	85.21 ± 0.07 e	99.56	*Agrobacterium salinitolerans*
SWLYDBP 39	75.45 ± 0.13 f	99.56	*Agrobacterium salinitolerans*
SWLYDBP 40	10.09 ± 3.75 y	99.27	*Brucella intermedia*
SWLYDBP 41	60.62 ± 8.04 k	99.78	*Brucella intermedia*
SWLYDBP 42	55.27 ± 6.24 o	99.41	*Brucella intermedia*
SWLYDBP 43	67.09 ± 7.59 g	99.55	*Brucella intermedia*
SWLYDBP 44	98.62 ± 0.002 ab	99.68	*Brucella lupini*
SWLYDBP 45	89.33 ± 0.41 c	99.43	*Brucella lupini*
SWLYDBP 46	69.62 ± 0.31 g	99.42	*Brucella lupini*
SWLYDBP 47	99.17 ± 0.01 a	99.65	*Brevundimonas bullata*
SWLYDBP 48	63.29 ± 5.41 i	99.58	*Acinetobacter radioresistens*
SWLYDBP 49	98.36 ± 0.06 b	99.65	*Acinetobacter radioresistens*
SWLYDBP 50	98.91 ± 0.01 ab	99.65	*Acinetobacter radioresistens*
SWLYDBP 51	99.90 ± 0.003 a	99.87	*Gordonia alkanivorans*

Table S4 Genes encoding esterase/hydrolase in SWLYDBP 51 strain genome.

NO.	Gene ID	NR functional annotation	NO.	Gene ID	NR functional annotation
1	gene0046	alpha/beta hydrolase	49	gene2093	esterase
2	gene0070	alpha/beta hydrolase	50	gene2244	alpha/beta hydrolase
3	gene0132	alpha/beta hydrolase	51	gene2252	alpha/beta fold hydrolase
4	gene0192	alpha/beta fold hydrolase	52	gene2322	alpha/beta hydrolase
5	gene0198	alpha/beta hydrolase	53	gene2420	alpha/beta hydrolase
6	gene0202	alpha/beta fold hydrolase	54	gene2567	alpha/beta fold hydrolase
7	gene0263	alpha/beta hydrolase	55	gene2572	hydrolase
8	gene0264	alpha/beta fold hydrolase	56	gene2685	alpha/beta hydrolase
9	gene0282	alpha/beta fold hydrolase	57	gene2694	alpha/beta hydrolase
10	gene0334	alpha/beta hydrolase	58	gene2723	alpha/beta hydrolase
11	gene0340	esterase	59	gene2854	alpha/beta hydrolase
12	gene0354	alpha/beta hydrolase	60	gene2882	alpha/beta hydrolase
13	gene0421	alpha/beta fold hydrolase	61	gene2946	alpha/beta hydrolase
14	gene0426	alpha/beta hydrolase	62	gene2947	alpha/beta hydrolase
15	gene0492	alpha/beta hydrolase	63	gene3098	alpha/beta hydrolase
16	gene0517	alpha/beta hydrolase	64	gene3265	alpha/beta hydrolase
17	gene0524	alpha/beta fold hydrolase	65	gene3270	alpha/beta hydrolase
18	gene0537	hydrolase	66	gene3277	alpha/beta fold hydrolase
19	gene0628	alpha/beta hydrolase	67	gene3361	alpha/beta hydrolase
20	gene0673	alpha/beta hydrolase	68	gene3362	alpha/beta hydrolase
21	gene0684	alpha/beta hydrolase	69	gene3364	alpha/beta hydrolase
22	gene0701	carboxylesterase/lipase	70	gene3450	alpha/beta fold hydrolase
23	gene0715	alpha/beta fold hydrolase	71	gene3492	alpha/beta fold hydrolase

Continued Table S4 Genes encoding esterase/hydrolase in SWLYDBP 51 strain genome.

NO.	Gene ID	NR functional annotation	NO.	Gene ID	NR functional annotation
24	gene0737	alpha/beta hydrolase	72	gene3648	alpha/beta hydrolase
25	gene0750	alpha/beta hydrolase	73	gene3747	alpha/beta hydrolase
26	gene0759	alpha/beta hydrolase	74	gene3801	alpha/beta hydrolase
27	gene0837	alpha/beta hydrolase	75	gene3842	alpha/beta fold hydrolase
28	gene0858	carboxylesterase/lipase	76	gene3871	alpha/beta hydrolase
29	gene0859	alpha/beta hydrolase	77	gene3872	alpha/beta hydrolase
30	gene1049	alpha/beta hydrolase	78	gene3910	alpha/beta hydrolase
31	gene1076	alpha/beta hydrolase	79	gene3911	alpha/beta hydrolase
32	gene1077	alpha/beta fold hydrolase	80	gene3927	alpha/beta fold hydrolase
33	gene1201	alpha/beta fold hydrolase	81	gene4039	alpha/beta hydrolase
34	gene1230	alpha/beta fold hydrolase	82	gene4127	alpha/beta hydrolase
35	gene1259	alpha/beta fold hydrolase	83	gene4166	alpha/beta hydrolase
36	gene1350	alpha/beta fold hydrolase	84	gene4218	alpha/beta hydrolase
37	gene1468	alpha/beta-hydrolase	85	gene4269	alpha/beta hydrolase
38	gene1488	alpha/beta hydrolase	86	gene4281	alpha/beta hydrolase
39	gene1541	alpha/beta fold hydrolase	87	gene4355	alpha/beta hydrolase
40	gene1561	alpha/beta hydrolase	88	gene4422	alpha/beta fold hydrolase
41	gene1584	alpha/beta hydrolase	89	gene4487	alpha/beta hydrolase
42	gene1785	alpha/beta hydrolase	90	gene4500	alpha/beta hydrolase
43	gene1865	alpha/beta hydrolase	91	gene4526	alpha/beta hydrolase
44	gene1910	esterase	92	gene4542	alpha/beta fold hydrolase
45	gene1930	alpha/beta hydrolase	93	gene4636	alpha/beta fold hydrolase
46	gene1932	alpha/beta hydrolase	94	pA_gene0068	alpha/beta fold hydrolase
47	gene2042	alpha/beta hydrolase	95	pB_gene0074	hydrolase
48	gene2060	alpha/beta fold hydrolase			

Table S5 Annotation of genes degrading PA and its intermediate metabolites in SWLYDBP strain 51 genome.

Gene ID	functional gene	KO Description	Homology (%)
Gene 2575	*phtAa*	phthalate 3,4-dioxygenase subunit alpha [EC:1.14.12.-]	100
Gene 2576	*phtAb*	phthalate 3,4-dioxygenase subunit beta [EC:1.14.12.-]	100
Gene 2578	*phtAc*	phthalate 3,4-dioxygenase ferredoxin component	100
Gene 2574			78.1
gene4563	*phtB*	phthalate 3,4-*cis*-dihydrodiol dehydrogenase [EC:1.3.1.-]	40.3
gene0308			43.9
gene2580	*phtC*	3,4-dihydroxyphthalate decarboxylase [EC:4.1.1.69]	100
gene2585	*pcaH*	protocatechuate 3,4-dioxygenase, beta subunit [EC:1.13.11.3]	100
gene2586	*pcaG*	protocatechuate 3,4-dioxygenase, alpha subunit [EC:1.13.11.3]	100
gene2587	*pcaB*	3-carboxy-*cis*,*cis*-muconate cycloisomerase [EC:5.5.1.2]	100
gene3909	*pcaC*	4-carboxymuconolactone decarboxylase [EC:4.1.1.44]	100
gene0654			100
gene2588	*pcaL*	3-oxoadipate enol-lactonase / 4-carboxymuconolactone decarboxylase [EC:3.1.1.24 4.1.1.44]	100

Continued Table S5 Annotation of genes degrading PA and its intermediate metabolites in SWLYDBP strain 51 genome

Gene ID	Functional gene	KO description	Homology (%)
Gene 1865			100
Gene 0750			100
Gene 1584	*pcaD*	3-oxoadipate enol-lactonase [EC:3.1.1.24]	100
Gene 2854			100
Gene 2947			100
Gene 3265			100
Gene 3871			100
Gene 3872	*pcaD*	3-oxoadipate enol-lactonase [EC:3.1.1.24]	100
Gene 4177			100
Gene 4281			100
Gene 4175	*pcaJ*	3-oxoacid CoA-transferase subunit B [EC:2.8.3.5]	50.7
Gene 4176	*pcaI*	3-oxoacid CoA-transferase subunit A [EC:2.8.3.5]	43.0
Gene 4024			30.5

Continued Table S5 Annotation of genes degrading PA and its intermediate metabolites in SWLYDBP strain 51 genome

Gene ID	Functional gene	KO description	Homology (%)
Gene 1463	*ligA*	protocatechuate 4,5-dioxygenase, alpha chain [EC: 1.13.11.8]	100
gene2568	*ligB*	protocatechuate 4,5-dioxygenase, beta chain [EC:1.13.11.8]	100
gene0406			53.7
gene4468	*ligC*	2-hydroxy-4-carboxymuconate semialdehyde hemiacetal dehydrogenase [EC: 1.1.1.312]	32.8
gene0164			30.8
gene4171	*benA-xylX*	benzoate/toluate 1,2-dioxygenase subunit alpha [EC:1.14.12.10 1.14.12.-]	100
gene4565			100
gene4172	*benB-xylY*	benzoate/toluate 1,2-dioxygenase subunit beta [EC:1.14.12.10 1.14.12.-]	100
gene4173	*benC-xylZ*	benzoate/toluate 1,2-dioxygenase reductase component [EC:1.18.1.-]	100
gene4173	*benD-xylL*	(1R,6S)-1,6-dihydroxycyclohexa-2,4-diene-1-carboxylate:NAD^+ oxidoreductase (decarboxylating) [EC:1.3.1.25]	56.8
gene4167	*catC*	muconolactone D-isomerase [EC:5.3.3.4]	100
gene4168	*catA*	catechol 1,2-dioxygenase [EC:1.13.11.1]	100

Continued Table S5 Annotation of genes degrading PA and its intermediate metabolites in SWLYDBP strain 51 genome.

Gene ID	Functional gene	KO description	Homology (%)
gene3421	*catB*	muconate cycloisomerase [EC:5.5.1.1]	100
gene4538			36.4
gene3442			100
gene2553	*fadA*	acetyl-CoA acyltransferase [EC:2.3.1.16]	100
gene2629			100
gene2662			100
gene2691			100
gene4426	*fadJ*	3-hydroxyacyl-CoA dehydrogenase / enoyl-CoA hydratase / 3-hydroxybutyryl-CoA epimerase [EC:1.1.1.35 4.2.1.17 5.1.2.3]	100
gene0662	*paaH*	3-hydroxybutyryl-CoA dehydrogenase [EC:1.1.1.157]	100
gene4137			100
gene0779	*paaF*	enoyl-CoA hydratase [EC:4.2.1.17]	100
gene1442			100
gene2555			100
gene4023			100

Continued Table S5 Annotation of genes degrading PA and its intermediate metabolites in SWLYDBP strain 51 genome.

Gene ID	Functional gene	KO description	Homology (%)
gene4180			100
gene0780	*mhpD*	2-keto-4-pentenoate hydratase [EC:4.2.1.80]	100
gene1358			100
gene1009	*hadAB*	meromycolic acid (3R)-3-hydroxyacyl-[acyl-carrier protein] dehydratase HadAB [EC:4.2.1.-]	100
gene2794			100
gene1178	*bcrC*	undecaprenyl-diphosphatase [EC:3.6.1.27]	100
gene1296	*badH*	2-hydroxycyclohexanecarboxyl-CoA dehydrogenase [EC:1.1.1.-]	100
gene2952			100
gene1313	*badA*	4-hydroxybenzoate-CoA ligase" [EC:6.2.1.27 6.2.1.25]	34.2
gene3437	*atoB*	acetyl-CoA C-acetyltransferase [EC:2.3.1.9]	100
gene0757			100
gene1361			100
gene2471			100
gene3035			100
gene3345			100
gene3434			100
gene4005			100
Gene 4006			100
Gene 4012			100
Gene 4031			100

Continued Table S5 Annotation of genes degrading PA and its intermediate metabolites in SWLYDBP strain 51 genome.

Gene ID	Functional gene	KO description	Homology (%）
Gene 4178			100
Gene 4220			100
Gene 4370			100
Gene 4427			100
Gene 4211	*GCDH*	glutaryl-CoA dehydrogenase [EC:1.3.8.6]	100
